# Inequities in COVID-19 morbidity in Brazil: the influence of demographic and socioeconomic characteristics and preexisting health conditions

**DOI:** 10.1590/1980-549720250056

**Published:** 2025-12-08

**Authors:** Célia Landmann Szwarcwald, Deborah Carvalho Malta, Wanessa da Silva Almeida, Paulo Roberto Borges de Souza, Giseli Nogueira Damacena, Crizian Saar Gomes, Euclides Ayres de Castilho

**Affiliations:** I Fundação Oswaldo Cruz, Institute of Scientific and Technological Communication and Information in Health, Health Information Laboratory – Rio de Janeiro (RJ), Brazil.; II Universidade Federal de Minas Gerais, School of Nursing – Belo Horizonte (MG), Brazil.; III Universidade de São Paulo, School of Medicine, Department of Preventive Medicine – São Paulo (SP), Brazil.

**Keywords:** COVID-19, Health surveys, Vaccination, Morbidity, Brazil

## Abstract

**Objective:**

To analyze COVID-19 morbidity according to sociodemographic characteristics and preexisting health conditions, based on data from a survey conducted in 2023 online called "ConVid-2 Behavior Survey".

**Methods:**

This was a cross-sectional epidemiological study using the Respondent-Driven Sampling (RDS) method. Prevalence estimates and 95% confidence intervals were calculated for five COVID-19-related indicators. Logistic regression models were applied to test the hypothesis of associations between outcomes and sociodemographic characteristics.

**Results:**

The sample included 3,805 individuals aged 18 years or older. Approximately 50% of participants had received four or more doses of the COVID-19 vaccine. The estimated prevalence of COVID-19 was 49.5%, with significant and increasing gradients by age and decreasing gradients by education level. Long COVID was identified in 32% of individuals with confirmed COVID-19, with the highest proportions among women (38.3%; OR=0.52; p=0.002), those with financial difficulties (38.9%; OR=1.68; p=0.02), and those with a chronic noncommunicable disease (36.3%; OR=1.58; p=0.03). The highest rate of hospitalization occurred among those with Long COVID (12.7%). Death of a household member due to COVID-19 was reported by 5.1% of participants.

**Conclusion:**

The findings revealed major socioeconomic inequalities across all indicators related to COVID-19 morbidity. Older age, preexisting health conditions, and Long COVID contributed to greater disease severity and increased hospitalization. These findings are relevant to inform public policies aimed at supporting the diagnosis and management of COVID-19-related complications within the public health system.

## INTRODUCTION

The COVID-19 pandemic has brought countless challenges to Brazilians in terms of both socioeconomic conditions and health^1^. The excessive number of deaths and the high mortality rate, one and a half times higher than the global mortality rate at the end of 2021^2^, highlighted the vulnerabilities of the healthcare system and the weakness of public policies to address the health crisis that ravaged the country^3–5^. The public health measures necessary to control the epidemic were adopted late, increasing the spread of the disease and resulting in numerous preventable deaths^6,7^.

A study of COVID-19 mortality in Brazil showed that the COVID-19 mortality rate was 1.5 per thousand inhabitants in 2020–21, accounting for approximately one-fifth of deaths from all causes during that period. In March 2021, when COVID-19 mortality rates peaked, the total number of COVID-19 deaths reached about four thousand deaths per day, a number higher than the daily average of deaths from all causes in 2019, before the pandemic arrived in the country^8^.

Regarding the analysis of COVID-19 mortality, the pandemic's unequal impact by educational level was also noted. Mortality rates showed a decreasing gradient by educational level, with a mortality rate 3.3 times higher among illiterate individuals than among those with higher education or more. The analysis of mortality rates at the subnational level also revealed significant regional differences, with higher mortality rates in the North and Central-West regions, once again revealing the stark regional and educational disparities in Brazil^9^.

The need to acquire greater knowledge about SARS-CoV-2 infection and other health problems directly or indirectly related to the pandemic has stimulated the use of the internet as a means of obtaining information quickly^10–13^. In Brazil, with the aim of investigating changes in the living and health conditions of the Brazilian population during the COVID-19 pandemic, the "ConVid: Behavior Survey" was carried out in a virtual environment in 2020. The information was collected through chain sampling, using the method known as virtual snowball sampling^14^.

The results of the ConVid: Behavioral Survey, conducted among the Brazilian adult population, showed that the guidance of home confinement had significant implications for the socioeconomic context, with a significant reduction in family income, especially in households with precarious living conditions^1^. Conversely, the restriction of physical contact between people, the loss of freedom of movement, and uncertainty about the disease provoked feelings of anguish, anxiety, and sadness, leading to sleep problems, depression, and other mental disorders^15–18^.

In 2023, driven by the need to gain knowledge about the health situation in the post-pandemic period, a new online survey was conducted, which was called "ConVid-2: Behavioral Survey." From July to December 2023, a self-administered online questionnaire was used, completed using a cell phone or computer with internet access. Respondent-driven sampling (RDS) was used to collect data, beginning with a fixed number of invitations containing a link to access the electronic questionnaire^19^.

The objective of this study was to analyze COVID-19 vaccination coverage and some aspects of COVID-19 morbidity, such as the prevalence of SARS-CoV-2 infection, the prevalence of Long COVID^20^, and the prevalence of hospitalization due to worsening of the disease. Additionally, the characteristics of households where a resident died from COVID-19 were analyzed.

## METHODS

### Study design

A cross-sectional epidemiological study conducted using chain sampling in a virtual environment among the Brazilian adult population (18 years or older) from July to December 2023, using the RDS methodology^19^.

The "ConVid-2: Behavioral Research" survey was developed by the Oswaldo Cruz Foundation, in partnership with the Federal University of Minas Gerais, State University of Campinas, Federal University of Ouro Preto, and Federal University of Sergipe.

The inclusion criteria for participation in the survey were being 18 years of age or older and residing in Brazil during the COVID-19 pandemic.

The project was approved by the National Research Ethics Committee on December 22, 2022, under protocol number 5.836.202. An online invitation to participate was sent, and before responding to the questionnaire, participants completed an informed consent form. All responses were anonymous and did not reveal any form of identification.

### Sample

The individual recruitment method was RDS, which uses a widely recognized statistical analysis methodology. RDS is considered a complex sampling method, with unequal selection probabilities and clusters composed of individuals recruited by the same participant. To estimate the variances of the indicators of interest, the bootstrap procedure was used through several simulations of samples generated with the same process that originated the total sample^21^.

To obtain a representative sample of the population according to sociodemographic characteristics, a post-stratification^22^ procedure was used on the basis of population estimates from the 2022 Continuous National Household Sample Survey (PNAD Contínua-2022) of the Brazilian Institute of Geography and Statistics^23^, by sex (male; female), age group (18–39; 40–59; 60+), level of education (incomplete high school or less; complete high school or incomplete higher education; complete higher education or more), and race/skin color (white; non-white)

The minimum sample size of 3,600 individuals aged 18 or older was calculated based on a simple random sample, assuming a design effect of 2, as suggested by Salganik and Heckathorn^24^. Further details on the use of the RDS method in a virtual environment and the information collection procedure were presented in another article from the ConVid-2 research^25^.

### Study variables

- Sociodemographic characteristics:
  - Sex (male, female);
  - Age range (18–39, 40–59, 60+);
  - Level of education (incomplete high school, complete high school or incomplete higher education, complete higher education or further);
  - Skin color (white, non-white);
  - Household income per capita (≤0.5, >0.5 and ≤1, >1 minimum wage);
  - Financial hardship (Are the residents of your household currently facing financial hardship? Yes or no;
  - Food insecurity (In the last three months, have you ever been concerned about not having enough money to buy food? Yes or no);
- Preexisting health conditions:
  - Self-rated health (very good/good, not good);
  - Self-reported medical diagnosis of some chronic noncommunicable disease (NCD) (yes or no);
- Indicators related to COVID-19:
  - Prevalence of SARS-CoV-2 infection (after reclassifying the unknowns, that is, those who did not know whether or not they had COVID-19);
  - Proportion (%) of individuals who received four or more doses of the COVID-19 vaccine;
  - Prevalence of Long COVID (proportion of people who had confirmed COVID-19 and the presence of symptoms and sequelae that persisted for three months or more after acute SARS-CoV-2 infection;
  - Prevalence of hospitalization (proportion of people who had confirmed COVID-19, required hospitalization, and received it);
  - Proportion of households in which a resident died from COVID-19.

### Analysis of data

In this study, prevalence of COVID-19-related indicators was estimated according to sociodemographic characteristics and preexisting health conditions. Logistic regression models were used to test the hypothesis of association between outcomes and sociodemographic characteristics. Significance levels lower than 5% were considered significant.

Statistical analysis was performed using the Statistical Package for the Social Sciences, version 21, complex samples module, taking into account the effects of the sampling design.

### Data availability statement:

Data available upon request.

## RESULTS

The study included 3,805 individuals aged 18 or older who responded to the electronic questionnaire between July and December 2023.

Table 1 presents the proportions of individuals who received four or more doses of the COVID-19 vaccine according to sociodemographic characteristics and preexisting health conditions. In Brazil, 49.9% of participants received four or more doses of the COVID-19 vaccine.

**Table 1:** Associations* of the proportion of individuals who received four or more doses of COVID-19 vaccine with demographic, socioeconomic characteristics and preexisting health conditions. Brazil, 2023.

Variables	Categories	% persons with four or more doses	OR	95%CI (OR)	p-value
Total		49.9	-	-	-
Sex	Male	43.3	0.618	0.457–0.837	0.002
	Female	55.2	1.000	-	-
Age range (years)	18–39	33.2	0.136	0.077–0.242	0.000
	40–59	55.6	0.343	0.180–0.653	0.001
	60 or older	78.5	1.000	-	-
Skin color	White	55.4	1.474	1.100–1.975	0.009
	Non-white	45.7	1.000	-	-
Level of education	Incomplete high school	49.1	0.528	0.352–0.794	0.002
	Complete high school	44.1	0.431	0.346–0.537	0.000
	Complete higher education	64.6	1.000	-	-
Household income per capita	≤0.5 MW	40.6	0.435	0.299–0.631	0.000
	>0.5 and ≤1 MW	44.7	0.513	0.371–0.711	0.000
	>1 MW	61.2	1.000	-	-
Financial difficulties	Yes	45.3	0.730	0.572–0.932	0.012
	No	53.1	1.000	-	-
Food insecurity	Yes	44.2	0.719	0.556–0.929	0.012
	No	52.4	1.000	-	-
Self-rated health	Not good	51.2	1.085	0.826–1.424	0.557
	Good	49.2	1.000	-	-
Some NCD	Yes	56.5	1.822	1.353–2.455	0.000
	No	41.7	1.000	-	-

* Measured by the ratio of the cross-products (odds ratio) of the logistic regression model of each variable; OR: odds ratio; 95%CI: 95% confidence interval; MW: minimum wage; NCD: chronic non-communicable disease.

Regarding demographic characteristics, the proportion was significantly lower among men (43.3%) compared to women (55.2%) (odds ratio — OR=0.618, p=0.002), among people aged 60 or over (78.5%) compared to those aged 18 to 39 (33.2%) (OR=0.136, p<0.01), among those with incomplete high school (49.1%) compared to those with complete higher education (64.6%) (OR=0.528, p=0.002), and among white individuals (55.4%) compared to non-white individuals (45.7%) (OR=1.474, p=0.009). Regarding socioeconomic inequalities, the proportion of individuals with four or more vaccine doses was significantly lower among those with an income below 0.5 minimum wages per capita (40.6%) compared to those with higher per capita household income (61.2%) (OR=0.435, p<0.01%). Significant differences were also observed for residents of households facing financial hardship (45.3%) and food insecurity (44.2%) compared to those without these problems (53.1 and 52.4%, respectively). Regarding preexisting health conditions, a significantly higher percentage was found only for individuals who reported a diagnosis of an NCD (56.5%) compared to others (41.7%).

In Brazil, the self-reported prevalence of COVID-19 was 46.7%, with 40.3% confirmed by laboratory testing and 6.4% unconfirmed. The proportion of people who were unable to say whether they had COVID-19 was large, 15.3%, making it difficult to estimate prevalence of SARS-CoV-2 infection. Therefore, a multivariate logistic regression model was used with the variables sex, age range, and health insurance coverage to reclassify those who were unknown.

Table 2 presents prevalence of SARS-CoV-2 infection after applying the multivariate logistic model to reclassify those who were unknown ("do not know if they had COVID-19"). According to the results of the statistical model, the estimated prevalence of COVID-19 was 49.5%. The analysis showed significant differences by age, with the highest proportions among individuals aged 40 or older (65.5%) compared to those under 40 (31%). Regarding differences by sex, men showed a significantly higher prevalence (67.5%) than women (33.3%). Regarding skin color, white individuals had a lower prevalence (43.3%) than non-white individuals (54.1%). Disparities by socioeconomic status were very pronounced, with higher COVID-19 prevalence rates among the poorest individuals, reaching proportions greater than 58% among those with lower levels of education and income, and among those facing financial hardship and lack of money to buy food (food insecurity). Also, among individuals who rated their health as poor, with an estimated COVID-19 prevalence of 57.3%. All differences were significant at the 1% level of significance, except for the presence of an NCD, which was not significant.

**Table 2:** Associations* of prevalence of COVID-19 with demographic, socioeconomic characteristics and preexisting health conditions. Brazil, 2023.

Variables	Categories	Prevalence (%) of COVID-19**	OR	95%CI (OR)	p-value
Total		49.5	-	-	-
Sex	Male	67.5	4.147	2.794–6.154	0.000
	Female	33.3	1.000	-	-
Age range (years)	18–39	31.0	0.236	0.168–0.332	0.000
	40 or older	65.5	1.000	-	-
Skin color	White	43.3	0.648	0.493–0.852	0.002
	Non-white	54.1	1.000	-	-
Level of education	Incomplete high school	66.6	3.535	2.259–5.534	0.000
	Complete high school or more	36.1	1.000	-	0.000
Household income per capita	≤0.5 MW	63.3	3.252	2.243–4.714	0.000
	>0.5 and ≤1 MW	54.7	2.281	1.533–3.395	0.000
	>1 MW	34.6	1.000	-	-
Financial difficulties	Yes	58.5	1.890	1.367–2.614	0.000
	No	42.8	1.000	-	-
Food insecurity	Yes	63.1	2.266	1.710–3.003	0.000
	No	43.0	1.000	-	-
Self-rated health	Not good	57.3	1.659	1.173–2.347	0.004
	Good	44.8	1.000	-	-
Some NCD	Yes	52.3	1.285	0.936–1.764	0.121
	No	46.0	1.000	-	-

* Measured by the ratio of the cross-products (odds ratio) of the logistic regression model of each variable;

** after classifying cases that responded that they did not know whether they had COVID-19 using a logistic regression model; OR: odds ratio; 95%CI: 95% confidence interval; MW: minimum wage; NCD: chronic non-communicable disease.

Regarding symptoms, disabilities, illnesses, or sequelae related to COVID-19 that persisted for three months or more (Long COVID), 32% of individuals with confirmed COVID-19 reported these problems (Table 3). Significant differences at the 5% level of significance were found for sex, with women experiencing Long COVID more frequently than men, as well as financial hardship (38.9%) and food insecurity (40%). The two indicators of preexisting health conditions, poor self-rated health and the presence of some NCD, were also significantly associated (p<0.05) with Long COVID, with prevalence rates of 47.4 and 36.3% and ORs of 2.933 and 1.583, respectively.

**Table 3:** Associations* of Long COVID prevalence** with demographic and socioeconomic characteristics and preexisting health conditions. Brazil, 2023.

Variables	Categories	Prevalence (%) of Long COVID**	OR	95%CI (OR)	p-value
Total		32.0	-	-	-
Sex	Male	24.4	0.521	0.342–0.794	0.002
	Female	38.3	1.000	-	-
Age range (years)	18–39	29.1	0.991	0.563–1.745	0.975
	40–59	37.0	1.420	0.714–2.826	0.317
	60 or older	29.3	1.000	-	-
Skin color	White	28.8	0.753	0.530–1.071	0.114
	Non-white	35.0	1.000	-	-
Level of education	Incomplete high school	33.7	1.324	0.843–2.078	0.222
	Complete high school	33.6	1.318	0.998–1.740	0.052
	Complete higher education	27.8	1.000	-	-
Household income per capita	≤0.5 MW	30.3	1.127	0.639–1.987	0.679
	>0.5 and ≤1 MW	33.0	1.272	0.839–1.929	0.256
	>1 MW	27.9	1.000	-	-
Financial difficulties	Yes	38.9	1.675	1.159–2.420	0.006
	No	27.5	1.000	-	-
Food insecurity	Yes	40.0	1.598	1.071–2.383	0.022
	No	29.4	1.000	-	-
Self-rate health	Not good	47.4	2.933	1.957–4.395	0.000
	Good	23.5	1.000	-	-
Some NCD	Yes	36.3	1.583	1.047–2.395	0.030
	No	26.5	1.000	-	-

* Measured by the ratio of the cross-products (odds ratio) of the logistic regression model for each variable;

** presence of symptoms and sequelae that persisted for three months or more after acute SARS-CoV-2 infection among individuals who had COVID-19 confirmed by laboratory tests; OR: odds ratio; 95%CI: 95% confidence interval; MW: minimum wage; NCD: chronic non-communicable disease.

The analysis of prevalence among individuals who required and obtained hospital admission due to COVID-19 according to sociodemographic characteristics is presented in Table 4. Among individuals who had COVID-19 confirmed by laboratory tests, 5.2% required and obtained hospitalization. The comparison of demographic and socioeconomic variables revealed significant inequalities (p<0.05) in the need for hospitalization by age group and level of education: 2.6% among individuals under 40 years of age and 11.1% among the older individuals; 8.5% among individuals with incomplete high school compared to those with complete higher education (2.5%). It is also worth noting that, among individuals who had Long COVID, the need for hospitalization was significantly higher, reaching the highest proportion of hospitalizations (12.7%, OR=9.045, p<0.01).

**Table 4:** Associations* of the proportion of individuals who needed and obtained hospitalization due to COVID-19** with demographic and socioeconomic characteristics and preexisting health conditions. Brazil, 2023.

Variables	Categories	% persons who managed to be hospitalized**	OR	95%CI (OR)	p-value
Total		5.2	-	-	-
Sex	Male	7.7	2.354	0.865–6.410	0.094
	Female	3.4	1.000	-	-
Age range (years)	18–39	2.6	0.212	0.057–0.780	0.020
	40–59	5.6	0.473	0.119–1.886	0.288
	60 or older	11.1	1.000	-	-
Skin color	White	5.6	1.169	0.423–3.228	0.762
	Non-white	4.9	1.000	-	-
Level of education	Incomplete high school	8.5	3.667	1.065–12.623	0.039
	Complete high school	4.9	2.030	1.323–3.115	0.001
	Complete higher education	2.5	1.000	-	-
Long COVID	Yes	12.7	9.045	3.567–22.937	0.000
	No	1.6	1.000	-	-

* Measured by the ratio of the cross-products (odds ratio) of the logistic regression model for each variable;

** proportion of individuals who required and underwent hospitalization among individuals with laboratory-confirmed COVID-19; OR: odds ratio; 95%CI: 95% confidence interval; MW: minimum wage; NCD: chronic non-communicable disease.

The percentage of people who reported that a resident of the household died from COVID-19 was 5.1% (Table 5). For the comparative analysis, only two household-related variables were used: per capita household income and number of household residents. The results showed a decreasing gradient with increasing income, ranging from 7.8 to 2.9%, corresponding to households with per capita income less than or equal to half the minimum wage and greater than one minimum wage, respectively. According to the analysis by number of residents, the greater the household crowding, the greater the chance that a resident of the household died from COVID-19. In households with four or more residents, a significantly higher percentage was found (8.3%).

**Table 5:** Associations* of the proportion of households in which a resident died from COVID-19 according to household income per capita and number of residents in the household. Brazil, 2023.

Variables	Categories	% households in which a resident died from COVID-19	OR	95%CI (OR)	p-value
Total		5.1	-	-	-
Household income per capita	≤0.5 MW	7.8	2.839	1.383–5.829	0.005
	>0.5 and ≤1 MW	6.0	2.146	1.016–4.532	0.045
	>1 MW	2.9	1.000	-	-
Number of residents in the household	1 to 3	3.6	0.410	0.207–0.812	0.011
	4+	8.3	1.000	-	-

* Measured by the ratio of the cross-products (odds ratio) of the logistic regression model of each variable; OR: odds ratio; 95%CI: 95% confidence interval; MW: minimum wage.

## DISCUSSION

Several COVID-19-related indicators were analyzed in this second stage of the ConVid-2 survey, such as vaccination coverage, the prevalence of COVID-19, long-COVID, and the need for hospitalization due to complications arising from the disease.

Regarding vaccination, in February 2022, the independent production program for COVID-19 vaccines was consolidated in the country, enabling the delivery of the doses needed to meet demand in 2022 and ensure the sustainability of mass vaccination, especially when new variants of SARS-CoV-2 emerged^26^. However, despite the availability of free vaccination in several basic health units throughout Brazil, only about 50% received four or more doses, resulting in significant demographic and socioeconomic inequalities in care to prevent the complications caused by COVID-19. The proportion of people who had COVID-19 confirmed by diagnostic testing corresponded to over 40% of Brazilians, while 6% of participants reported having had SARS-CoV-2 infection, but without confirmation by laboratory tests. Furthermore, the results showed that 15.3% did not know if they had COVID-19, mainly among people with precarious socioeconomic conditions and who likely had difficulty accessing testing through the Unified Health System (SUS)^6,7^. After reclassifying the unknown cases using a multivariate logistic regression model, the estimated prevalence of COVID-19 was 49.5%. Increasing gradients were found for age and decreasing gradients for education level, in similarity to COVID-19 mortality^8^.

Long COVID was identified in 32% of individuals with confirmed COVID-19, or 12.8% of the total participants, a figure close to the 10% estimate cited in the literature^27^. Associated with preexisting health conditions, such as poor self-rated health and a previous diagnosis of an NCD, Long COVID resulted in significant long-term consequences, limiting daily activities and affecting quality of life, as evidenced in Brazil and other countries^28,29^.

Prevalence of Long COVID estimated in the survey was higher among women and among individuals with NCDs, such as reported in an article conducted in southern Brazil^30^. The findings of this article showed that having had long COVID was associated with the need for hospitalization, in agreement with the findings of another Brazilian study^31^.

Financial difficulties, as well as food insecurity, were important factors in COVID-19 morbidity. During the pandemic, there were significant losses in family income and employment status^1^, which have likely not yet been fully recovered. Furthermore, the results of this survey revealed significant socioeconomic inequalities for all COVID-19 morbidity indicators considered in the survey. Thus, in addition to the increase in the proportion of people with precarious living conditions during the pandemic, individuals experiencing financial hardship were the least vaccinated and had the highest prevalence of SARS-CoV-2 infection and Long COVID, which, in turn, resulted in an increase in the proportion of individuals requiring more healthcare.

Among individuals with confirmed COVID-19, 5.2% required hospitalization, placing a strain on hospitals and intensive care units. Having had Long COVID was the variable most associated with the need for hospitalization. These results reinforce the importance of identifying and treating Long COVID and optimizing the allocation of resources within the SUS for specific care actions dedicated to treating Long COVID in public health services^31,32^.

Regarding the death of a household member, an association was found with households with lower per capita income and greater household overcrowding, reflecting the unequal impact of the pandemic on socially disadvantaged Brazilian families^33^. These families, however, also have a higher number of residents per room, increasing the risk of SARS-CoV-2 transmission.

In conclusion, the results of this study showed that the health situation in Brazil was significantly affected by the COVID-19 pandemic in terms of both mortality and morbidity, especially among the most disadvantaged population. The findings revealed significant socioeconomic inequalities, with the lowest vaccination rates and the highest prevalence of COVID-19 among individuals with lower incomes, those with greater financial difficulties, and those experiencing food insecurity. Advanced age and preexisting health conditions (such as the presence of a chronic disease) influenced the severity of the disease and the increase in hospitalizations. Among individuals who reported long-COVID, some health problems persist to the present, such as worsening health perception and limitations in usual activities. In this context, the availability of timely information in the post-pandemic period presented in this study is crucial to inform public policies aimed at diagnosing and managing COVID-19-related complications in the public health system.

Among the main limitations of online chain sampling surveys are: people without internet access have zero probability of being selected; and individuals with low levels of education and the elderly often have significant difficulty completing the online questionnaire, disrupting the development of networks. In virtual RDS sampling with peer recruitment, since participants are volunteers, neither the probabilities of selection nor the non-response rate can be estimated.

Another challenge is obtaining a representative sample of the Brazilian population. In this study, a post-stratification procedure was used based on population estimates from the 2022 Continuous National Household Sample Survey (PNAD Contínua), and the distributions of the variables used were, in fact, very similar to those of PNAD. However, the exclusion of a variable associated with one of the outcomes considered in the post-stratification procedure can cause errors in the mean estimates of these outcomes.

Unlike the 2020 survey using a virtual snowball method^14^, the inclusion of recruiter-recruitee pairs allowed for the estimation of variances of the indicators of interest and confidence intervals, as well as for the performance of hypothesis tests with greater reliability.
