# Supplementary material for: Inequities in COVID-19 morbidity in Brazil: the influence of demographic and socioeconomic characteristics and preexisting health conditions
Source: Rev Bras Epidemiol. 2025 Dec 8;28:e250056. doi: 10.1590/1980-549720250056 (PMC12685276; doi:10.1590/1980-549720250056)
Supplement: Translation

# Translation: Iniquidades da morbidade por COVID-19 no Brasil: a influência das características demográficas, socioeconômicas e condições de saúde preexistentes

**DOI:** 10.1590/1980-549720250056.2

**Authors:** Célia Landmann Szwarcwald, Deborah Carvalho Malta, Wanessa da Silva Almeida, Paulo Roberto Borges de Souza, Giseli Nogueira Damacena, Crizian Saar Gomes, Euclides Ayres de Castilho

**Affiliations:** I Fundação Oswaldo Cruz, Instituto de Comunicação e Informação Científica e Tecnológica em Saúde, Laboratório de Informação em Saúde – Rio de Janeiro (RJ), Brasil.; II Universidade Federal de Minas Gerais, Escola de Enfermagem – Belo Horizonte (MG), Brasil.; III Universidade de São Paulo, Faculdade de Medicina, Departamento de Medicina Preventiva – São Paulo (SP), Brasil.

**Keywords:** COVID-19, Inquéritos epidemiológicos, Vacinação, Morbidade, Brasil

## Abstract

**Objetivo:**

Analisar a morbidade por COVID-19 segundo as características sociodemográficas e condições de saúde preexistentes, com base nos dados de um inquérito realizado em 2023 pela internet denominado de "ConVid-2: Pesquisa de Comportamentos".

**Métodos:**

Estudo transversal epidemiológico realizado pelo método *respondent driven sampling* (RDS). Foram estimados as prevalências e os respectivos intervalos de confiança de cinco indicadores relacionados à COVID-19. Para testar a hipótese de associação dos desfechos com as características sociodemográficas, foram utilizados modelos de regressão logística.

**Resultados:**

O total da amostra foi de 3.805 indivíduos de 18 anos ou mais. Aproximadamente, 50% dos participantes tomaram quatro doses ou mais de vacina antiCOVID-19. A prevalência estimada de COVID-19 foi de 49,5%, com gradientes significativos e crescentes para a idade e decrescentes por grau de instrução. A COVID-longa foi identificada em 32% dos indivíduos que tiveram COVID-19 confirmada, com as maiores proporções entre as mulheres (38,3%; *odds ratio* — OR=0,52; p=0,002), com dificuldades financeiras (38,9%; OR=1,68; p=0,02) e com alguma doença crônica não transmissível (36,3%; OR=1,58; p=0,03). O maior percentual de internação hospitalar ocorreu entre os que tiveram COVID-longa (12,7%). O falecimento de algum morador do domicílio por COVID-19 foi relatado por 5,1% dos participantes.

**Conclusão:**

Os achados revelaram grandes desigualdades socioeconômicas em todos os indicadores relacionados à morbidade por COVID-19. A idade avançada, as condições preexistentes de saúde e a COVID-longa interferiram na gravidade da doença e no aumento de internações. Esses achados são relevantes para fornecer subsídios às políticas públicas dirigidas aos cuidados dedicados ao diagnóstico e manejo das complicações relacionadas à COVID-19 no sistema público de saúde.

## INTRODUÇÃO

A pandemia da COVID-19 trouxe inúmeros problemas aos brasileiros, tanto no que se refere às condições socioeconômicas como também na situação de saúde^1^. O excessivo número de mortes e a alta letalidade, uma vez e meia maior do que a letalidade global no fim de 2021^2^, evidenciaram as vulnerabilidades do sistema de saúde e a fragilidade das políticas públicas para o enfrentamento da crise sanitária que assolou o país^3–5^. As medidas de saúde pública necessárias para o controle da epidemia foram adotadas tardiamente, aumentando a disseminação da doença e implicando em diversas mortes evitáveis^6,7^.

Estudo de mortalidade por COVID-19 no Brasil mostrou que a taxa de mortalidade por COVID-19 foi de 1,5 por mil habitantes nos anos 2020–21, correspondendo a cerca de um quinto dos óbitos por todas as causas nesse período. Em março de 2021, quando ocorreu o pico das taxas de mortalidade por COVID-19, o número total de mortes por COVID-19 alcançou cerca de quatro mil mortes por dia, número superior à média diária de mortes por todas as causas em 2019, antes da chegada da pandemia no país^8^.

Ainda em relação à análise de mortalidade por COVID-19, constatou-se o impacto desigual da pandemia por grau de instrução. As taxas de mortalidade mostraram um gradiente decrescente por nível de escolaridade, atingindo uma taxa de mortalidade 3,3 vezes maior entre os indivíduos analfabetos do que aqueles com nível de escolaridade superior ou mais. A análise das taxas de mortalidade em níveis subnacionais revelou, igualmente, diferenças regionais significativas, com maiores taxas de mortalidade nas regiões Norte e Centro-Oeste, revelando uma vez mais as acentuadas disparidades regionais e por escolaridade no Brasil^9^.

A necessidade de aquisição de maior conhecimento sobre a infecção pelo SARS-Cov-2 e de outros problemas de saúde relacionados direta ou indiretamente à pandemia estimulou o uso da internet como meio para a obtenção de informações de forma ágil^10–13^. No Brasil, com a finalidade de investigar as mudanças nas condições de vida e de saúde da população brasileira durante a pandemia de COVID-19, foi realizada a "ConVid: Pesquisa de Comportamentos" em ambiente virtual no ano de 2020. As informações foram coletadas por amostragem em cadeia, pelo método conhecido como bola de neve virtual^14^.

Os resultados da ConVid: Pesquisa de Comportamentos realizada na população adulta brasileira mostraram que a orientação do confinamento domiciliar teve implicações relevantes no contexto socioeconômico, com redução significativa da renda familiar principalmente nos domicílios com condições de vida precárias^1^. Em contrapartida, a restrição de contato físico entre as pessoas, a perda da liberdade de ir e vir e as incertezas sobre a doença provocaram sentimentos de angústia, ansiedade e tristeza, gerando problemas no sono, depressão e outros transtornos mentais^15–18^.

No ano de 2023, incentivados pela necessidade de aquisição de conhecimento da situação de saúde no período pós-pandêmico, foi realizado novo inquérito pela internet, denominado de "ConVid-2: Pesquisa de Comportamentos". No período de julho a dezembro de 2023, utilizou-se um questionário virtual, autopreenchido por meio de celular ou computador com acesso à internet. Para a coleta de informações, foi utilizado o método de amostragem *respondente-driven sampling* (RDS), iniciando-se com o envio de um número fixo de convites com o *link* de acesso ao questionário eletrônico^19^.

O objetivo do presente trabalho foi analisar a cobertura de vacinação antiCOVID-19 e alguns aspectos da morbidade por COVID-19, tais como a prevalência da infecção pelo SARS-CoV-2, a prevalência de COVID-Longa^20^ e a prevalência de internação pelo agravamento da doença. Adicionalmente, foram analisadas as características dos domicílios em que houve morte de algum morador por COVID-19.

## MÉTODOS

### Desenho do estudo

Estudo transversal epidemiológico realizado por amostragem em cadeia em ambiente virtual na população adulta brasileira (18 anos ou mais) no período de julho a dezembro de 2023, utilizando a metodologia RDS^19^.

A pesquisa "ConVid-2: Pesquisa de Comportamentos" foi desenvolvida pela Fundação Oswaldo Cruz, em parceria com a Universidade Federal de Minas Gerais, a Universidade Estadual de Campinas, a Universidade Federal de Ouro Preto e a Universidade Federal de Sergipe.

Os critérios de inclusão para participação na pesquisa foram ter 18 anos ou mais de idade e residir no território brasileiro durante a pandemia de COVID-19.

O projeto foi aprovado pela Comissão Nacional de Ética em Pesquisa em 22 de dezembro de 2022, com número do protocolo 5.836.202. Foi enviado convite *on-line* para participação, e, antes de responder ao questionário, os participantes preencheram o Termo de Consentimento Livre e Esclarecido. Todas as respostas foram anônimas e sem nenhum tipo de identificação.

### Amostra

O método de recrutamento dos indivíduos foi o RDS, que dispõe de metodologia de análise estatística amplamente reconhecida. O RDS é considerado um método de amostragem complexo, com probabilidades desiguais de seleção e conglomerados compostos de pessoas recrutadas pelo mesmo participante. Para a estimação das variâncias dos indicadores de interesse, foi utilizado o procedimento de *bootstrap*, ou seja, várias simulações de amostras geradas com o mesmo processo originaram a amostra total^21^.

Para obter uma amostra representativa da população de acordo com as características sociodemográficas, utilizou-se um procedimento de pós-estratificação^22^ com base nas estimativas populacionais da Pesquisa Nacional por Amostra de Domicílios Contínua, do Instituto Brasileiro de Geografia e Estatística, de 2022 (PNAD Contínua-2022)^23^, por sexo (masculino; feminino), faixa etária (18–39; 40–59; 60+ anos), grau de escolaridade (ensino médio incompleto ou menos; ensino médio completo ou superior incompleto; superior completo ou mais) e raça/cor da pele (branca; não branca).

O cálculo do tamanho mínimo de amostra, de 3.600 pessoas com 18 anos ou mais, foi feito com base em uma amostra aleatória simples, considerando-se um efeito de desenho 2, conforme sugerido por Salganik e Heckathorn^24^. Maior detalhamento sobre a utilização do método RDS em ambiente virtual e sobre o procedimento de coleta de informações foi apresentado em outro artigo da pesquisa ConVid-2^25^.

### Variáveis do estudo

- Características sociodemográficas:
  - Sexo (masculino, feminino);
  - Faixa de idade (18–39, 40–59, 60+);
  - Grau de instrução (médio incompleto, médio completo ou superior incompleto, superior completo ou mais);
  - Cor da pele (branca, não branca);
  - Renda domiciliar *per capita* (<=0,5 salário mínimo, >0,5 e <=1 salário mínimo, >1);
  - Dificuldades financeiras (atualmente, os moradores do seu domicílio enfrentam dificuldades financeiras? Sim ou não;
  - Insegurança alimentar (nos últimos três meses, alguma vez no seu domicílio houve a preocupação que faltasse dinheiro para comprar comida? Sim ou não);
- Condições de saúde preexistentes:
  - Autoavaliação de saúde (muito boa/boa, não boa);
  - Diagnóstico médico autorreferido de alguma doença crônica não transmissível (DCNT) (sim ou não);
- Indicadores relacionados à COVID-19:
  - Prevalência da infecção por SARS-Cov-2 (após reclassificação dos ignorados, isto é, dos que não sabiam se haviam tido ou não COVID-19);
  - Proporção (%) de indivíduos que tomaram quatro ou mais doses de vacina contra COVID-19;
  - Prevalência de COVID-longa (proporção de pessoas que tiveram COVID-19 confirmada e presença de sintomas e sequelas que persistiram por três meses ou mais após a infecção aguda por SARS-CoV-2);
  - Prevalência de internação (proporção de pessoas que tiveram COVID-19 confirmada, precisaram da internação e a conseguiram);
  - Proporção de domicílios em que houve morte por COVID-19 de algum morador do domicílio.

### Análise dos dados

No presente estudo, foram estimadas as prevalências dos indicadores relacionados à COVID-19 segundo as características sociodemográficas e condições preexistentes de saúde. Para testar a hipótese de associação dos desfechos com as características sociodemográficas, foram utilizados modelos de regressão logística. Valores do nível de significância menores do que 5% foram considerados significativos.

A análise estatística foi feita pelo *software* Statistical Package for the Social Sciences, versão 21, módulo amostras complexas, considerando-se os efeitos do plano de amostragem.

### Declaração de disponibilidade de dados:

Data available upon request.

## RESULTADOS

Foram incluídas no estudo 3.805 pessoas de 18 anos ou mais de idade que responderam ao questionário eletrônico no período de julho a dezembro de 2023.

Na Tabela 1, são apresentadas as proporções de indivíduos que tomaram quatro doses ou mais contra COVID-19 segundo as características sociodemográficas e condições de saúde preexistentes. No Brasil, 49,9% dos participantes tomaram quatro doses ou mais de vacina antiCOVID-19.

**Tabela 1:** Associação* da proporção de indivíduos que tomaram quatro doses ou mais de vacina contra COVID-19 com as características demográficas, socioeconômicas e condições de saúde preexistentes. Brasil, 2023.

Variáveis	Categorias	% pessoas com quatro ou mais doses	OR	IC95% (OR)	Valor p
Total		49,9	-	-	-
Sexo	Masculino	43,3	0,618	0,457–0,837	0,002
	Feminino	55,2	1,000	-	-
Faixa de idade (anos)	18–39	33,2	0,136	0,077–0,242	0,000
	40–59	55,6	0,343	0,180–0,653	0,001
	60 ou mais	78,5	1,000	-	-
Cor da pele	Branca	55,4	1,474	1,100–1,975	0,009
	Não branca	45,7	1,000	-	-
Grau de instrução	Médio incompleto	49,1	0,528	0,352–0,794	0,002
	Médio completo	44,1	0,431	0,346–0,537	0,000
	Superior completo	64,6	1,000	-	-
Renda domiciliar *per capita*	≤0,5 SM	40,6	0,435	0,299–0,631	0,000
	>0,5 e ≤1 SM	44,7	0,513	0,371–0,711	0,000
	>1 SM	61,2	1,000	-	-
Dificuldades financeiras	Sim	45,3	0,730	0,572–0,932	0,012
	Não	53,1	1,000	-	-
Insegurança alimentar	Sim	44,2	0,719	0,556–0,929	0,012
	Não	52,4	1,000	-	-
Autoavaliação de saúde	Não boa	51,2	1,085	0,826–1,424	0,557
	Boa	49,2	1,000	-	-
Alguma DCNT	Sim	56,5	1,822	1,353–2,455	0,000
	Não	41,7	1,000	-	-

* Medida pela razão dos produtos cruzados (*odds ratio*) do modelo de regressão logística de cada variável; OR: *odds ratio*; IC95%: intervalo de confiança de 95%; SM: salário mínimo; DCNT: doença crônica não transmissível.

Em relação às características demográficas, a proporção foi significativamente menor entre os homens (43,3%) em relação às mulheres (55,2%) (*odds ratio* — OR=0,618, p=0,002), entre as pessoas com 60 anos ou mais (78,5%) em comparação às de 18 a 39 anos (33,2%) (OR=0,136, p<1%), entre as que têm grau de instrução médio incompleto (49,1%) em relação a superior completo (64,6%) (OR=0,528, p=0,002) e entre os indivíduos de cor branca (55,4%) quando comparados aos de cor não branca (45,7%) (OR=1,474, p=0,009).

No que se refere às desigualdades socioeconômicas, a proporção de indivíduos com quatro doses ou mais de vacina foi significativamente menor entre aqueles com renda inferior a 0,5 salário mínimo *per capita* (40,6%) quando comparados aos de maior rendimento domiciliar *per capita* (61,2%) (OR=0,435, p<1%). Diferenças significativas foram obtidas, igualmente, para os moradores de domicílios que enfrentam dificuldades financeiras (45,3%) e insegurança alimentar (44,2%) comparados aos que não enfrentam esses problemas (53,1 e 52,4%, respectivamente). Quanto às condições de saúde preexistentes, percentual significativamente maior foi encontrado apenas para os indivíduos que apontaram diagnóstico de alguma DCNT (56,5%) em relação aos demais (41,7%).

No Brasil, a prevalência de COVID-19 autorrelatada foi de 46,7%, sendo 40,3% confirmada por teste laboratorial e 6,4% não confirmada. A proporção de pessoas que não souberam dizer se tiveram COVID-19 ou não foi grande, 15,3%, dificultando a estimação da prevalência de infecção por SARS-CoV-2. Sendo assim, foi utilizado um modelo de regressão logística multivariado com as variáveis sexo, faixa de idade e posse de plano de saúde para reclassificação dos ignorados.

Na Tabela 2, é apresentada a prevalência da infecção por SARS-CoV-2, após a aplicação do modelo logístico multivariado para reclassificar os ignorados ("não sabem se tiveram COVID-19"). De acordo com os resultados do modelo estatístico, a estimativa da prevalência de COVID-19 foi de 49,5%. A análise mostrou diferenças significativas por idade, com as maiores proporções entre os indivíduos com 40 anos ou mais (65,5%) em relação aos com menos de 40 anos de idade (31%). No que se refere às diferenças por sexo, os homens mostraram prevalência significativamente maior (67,5%) do que as mulheres (33,3%). Quanto à cor da pele, os indivíduos de cor branca apresentaram menor prevalência (43,3%) do que os de cor não branca (54,1%).

**Tabela 2:** Associações* da prevalência de COVID-19 com as características demográficas, socioeconômicas e condições de saúde preexistentes. Brasil, 2023.

Variáveis	Categorias	Prevalência (%) de COVID-19**	OR	IC95% (OR)	Valor p
Total		49,5	-	-	-
Sexo	Masculino	67,5	4,147	2,794–6,154	0,000
	Feminino	33,3	1,000	-	-
Faixa de idade (anos)	18–39	31,0	0,236	0,168–0,332	0,000
	40 ou mais	65,5	1,000	-	-
Cor da pele	Branca	43,3	0,648	0,493–0,852	0,002
	Não branca	54,1	1,000	-	-
Grau de instrução	Médio incompleto	66,6	3,535	2,259–5,534	0,000
	Médio completo ou mais	36,1	1,000	-	0,000
Renda domiciliar *per capita*	≤0,5 SM	63,3	3,252	2,243–4,714	0,000
	>0,5 e ≤1 SM	54,7	2,281	1,533–3,395	0,000
	>1 SM	34,6	1,000	-	-
Dificuldades financeiras	Sim	58,5	1,890	1,367–2,614	0,000
	Não	42,8	1,000	-	-
Insegurança alimentar	Sim	63,1	2,266	1,710–3,003	0,000
	Não	43,0	1,000	-	-
Autoavaliação de saúde	Não boa	57,3	1,659	1,173–2,347	0,004
	Boa	44,8	1,000	-	-
Alguma DCNT	Sim	52,3	1,285	0,936–1,764	0,121
	Não	46,0	1,000	-	-

* Medida pela razão dos produtos cruzados (*odds ratio*) do modelo de regressão logística de cada variável;

** após classificação dos casos que responderam que não sabiam se tinham tido COVID-19 por modelo de regressão logística; OR: *odds ratio*; IC95%: intervalo de confiança de 95%; SM: salário mínimo; DCNT: doença crônica não transmissível.

As disparidades por nível socioeconômico foram muito acentuadas, com maiores prevalências de COVID-19 entre os indivíduos mais carentes, alcançando proporções maiores do que 58% entre os de menor nível de instrução e rendimento e entre aqueles que enfrentam dificuldades financeiras e problemas de falta de dinheiro para comprar comida (insegurança alimentar), bem como entre os indivíduos que avaliaram sua saúde como não boa, com prevalência estimada de COVID-19 de 57,3%. Todas as diferenças foram significativas no nível de 1% de significância, exceto para a presença de alguma DCNT que não foi significativa.

No tocante aos sintomas, às incapacidades, às doenças ou às sequelas relacionadas à COVID-19 que persistiram por três meses ou mais (COVID-Longa), 32% dos indivíduos que tiveram COVID-19 confirmada relataram esses problemas (Tabela 3). Diferenças significativas no nível de 5% de significância foram encontradas para sexo, com as mulheres tendo COVID-longa com maior frequência do que os homens, como também dificuldades financeiras (38,9%) e insegurança alimentar (40%). Os dois indicadores de condições preexistentes de saúde, autoavaliação de saúde não boa e presença de alguma DCNT, foram também significativamente associados (p<5%) à COVID-longa, com prevalências de 47,4 e 36,3% e OR de 2,933 e 1,583, respectivamente.

**Tabela 3:** Associações* da prevalência de COVID-longa** com as características demográficas, socioeconômicas e condições de saúde preexistentes. Brasil, 2023.

Variáveis	Categorias	Prevalência (%) de COVID-longa**	OR	IC95% (OR)	Valor p
Total		32,0	-	-	-
Sexo	Masculino	24,4	0,521	0,342–0,794	0,002
	Feminino	38,3	1,000	-	-
Faixa de idade (anos)	18–39	29,1	0,991	0,563–1,745	0,975
	40–59	37,0	1,420	0,714–2,826	0,317
	60 ou mais	29,3	1,000	-	-
Cor da pele	Branca	28,8	0,753	0,530–1,071	0,114
	Não branca	35,0	1,000	-	-
Grau de instrução	Médio incompleto	33,7	1,324	0,843–2,078	0,222
	Médio completo	33,6	1,318	0,998–1,740	0,052
	Superior completo	27,8	1,000	-	-
Renda domiciliar *per capita*	≤0,5 SM	30,3	1,127	0,639–1,987	0,679
	>0,5 e ≤1 SM	33,0	1,272	0,839–1,929	0,256
	>1 SM	27,9	1,000	-	-
Dificuldades financeiras	Sim	38,9	1,675	1,159–2,420	0,006
	Não	27,5	1,000	-	-
Insegurança alimentar	Sim	40,0	1,598	1,071–2,383	0,022
	Não	29,4	1,000	-	-
Autoavaliação de saúde	Não boa	47,4	2,933	1,957–4,395	0,000
	Boa	23,5	1,000	-	-
Alguma DCNT	Sim	36,3	1,583	1,047–2,395	0,030
	Não	26,5	1,000	-	-

* Medida pela razão dos produtos cruzados (*odds ratio*) do modelo de regressão logística de cada variável;

** presença de sintomas e sequelas que persistiram por três meses ou mais após a infecção aguda por SARS-CoV-2 entre os indivíduos que tiveram COVID-19 confirmada por testes laboratoriais; OR: *odds ratio*; IC95%: intervalo de confiança de 95%; SM: salário mínimo; DCNT: doença crônica não transmissível.

A análise da prevalência entre os indivíduos que precisaram de internação hospitalar pelo agravo da COVID-19 e a conseguiram segundo as características sociodemográficas está apresentada na Tabela 4. Entre as pessoas que tiveram COVID-19 confirmada por testes laboratoriais, 5,2% precisaram da internação e a conseguiram. A comparação das variáveis demográficas e socioeconômicas mostrou desigualdades significativas (p<5%) na necessidade de internação por faixa de idade e grau de escolaridade: 2,6% entre indivíduos com menos de 40 anos e 11,1% entre os idosos; 8,5% dos indivíduos com grau de instrução médio incompleto em comparação àqueles com ensino superior completo (2,5%). Interessa notar ainda que, entre os indivíduos que tiveram COVID-longa, a necessidade de internação foi significativamente maior, alcançando a maior proporção de internação (12,7%, OR=9,045, p<1%).

**Tabela 4:** Associações* da proporção de indivíduos que precisaram de internação por causa da COVID-19 e a conseguiram** com as características demográficas, socioeconômicas e condições de saúde preexistentes. Brasil, 2023.

Variáveis	Categorias	% pessoas que conseguiram internação**	OR	IC95% (OR)	Valor p
Total		5,2	-	-	-
Sexo	Masculino	7,7	2,354	0,865–6,410	0,094
	Feminino	3,4	1,000	-	-
Faixa de idade (anos)	18–39	2,6	0,212	0,057–0,780	0,020
	40–59	5,6	0,473	0,119–1,886	0,288
	60 ou mais	11,1	1,000	-	-
Cor da pele	Branca	5,6	1,169	0,423–3,228	0,762
	Não branca	4,9	1,000	-	-
Grau de instrução	Médio incompleto	8,5	3,667	1,065–12,623	0,039
	Médio completo	4,9	2,030	1,323–3,115	0,001
	Superior completo	2,5	1,000	-	-
COVID-Longa	Sim	12,7	9,045	3,567–22,937	0,000
	Não	1,6	1,000	-	-

* Medida pela razão dos produtos cruzados (*odds ratio*) do modelo de regressão logística de cada variável;

** proporção de indivíduos que precisaram de internação e a conseguiram entre os indivíduos que tiveram COVID-19 confirmada por testes laboratoriais; OR: *odds ratio*; IC95%: intervalo de confiança de 95%; SM: salário mínimo; DCNT: doença crônica não transmissível.

O percentual de pessoas que relataram que algum morador do domicílio faleceu por COVID-19 foi de 5,1% (Tabela 5). Para a análise comparativa, foram utilizadas apenas duas variáveis referentes ao domicílio: rendimento domiciliar *per capita* e número de moradores do domicílio. Os resultados mostraram um gradiente decrescente com o aumento da renda, com variação de 7,8 a 2,9%, correspondendo aos domicílios com rendimento *per capita* menor ou igual a meio salário mínimo e maior do que um salário mínimo, respectivamente. De acordo com a análise por número de moradores, quanto maior a aglomeração domiciliar, maior a chance de algum morador do domicílio ter falecido por COVID-19. Nos domicílios com quatro ou mais moradores, foi encontrado percentual significativamente maior (8,3%).

**Tabela 5:** Associações* da proporção de domicílios em que algum morador faleceu por COVID-19 segundo a renda domiciliar per capita e o número de moradores no domicílio. Brasil, 2023.

Variáveis	Categorias	% domicílios em que algum morador faleceu por COVID-19	OR	IC95% (OR)	Valor p
Total		5,1	-	-	-
Renda domiciliar *per capita*	≤0,5 SM	7,8	2,839	1,383–5,829	0,005
	>0,5 e ≤1 SM	6,0	2,146	1,016–4,532	0,045
	>1 SM	2,9	1,000	-	-
Número de moradores do domicílio	1 a 3	3,6	0,410	0,207–0,812	0,011
	4+	8,3	1,000	-	-

* Medida pela razão dos produtos cruzados (*odds ratio*) do modelo de regressão logística de cada variável; OR: *odds ratio*; IC95%: intervalo de confiança de 95%; SM: salário mínimo.

## DISCUSSÃO

Vários indicadores relacionados à COVID-19 puderam ser analisados nessa segunda etapa do inquérito ConVid-2, como a cobertura vacinal, as prevalências de COVID-19, de COVID-longa e de necessidade de internação por causa das complicações decorrentes da doença.

No que se refere à vacinação, em fevereiro de 2022, o programa de produção independente de vacinas contra a COVID-19 foi consolidado no país, possibilitando a entrega das doses necessárias para atender às demandas de 2022 e prover sustentabilidade da vacinação em massa, sobretudo quando surgiram novas variantes do SARS-CoV-2^26^. Contudo, apesar da disponibilidade de vacinação gratuita em várias unidades básicas de saúde em todo o território brasileiro, apenas cerca de 50% tomaram quatro doses ou mais, sendo encontradas grandes desigualdades demográficas e socioeconômicas nos cuidados para prevenir os agravos causados pela COVID-19.

A proporção de pessoas que tiveram COVID-19 confirmada por teste de diagnóstico correspondeu a mais de 40% de brasileiros, enquanto 6% dos participantes relataram ter tido a infecção por SARS-COV-2, mas sem confirmação por testes laboratoriais. Além disso, os resultados mostraram que 15,3% não sabiam se tiveram COVID-19, principalmente entre as pessoas com condições socioeconômicas precárias e que, provavelmente, tiveram dificuldades de acesso à testagem no Sistema Único de Saúde (SUS)^6,7^. Após a reclassificação dos casos ignorados por meio de um modelo de regressão logística multivariada, a prevalência estimada de COVID-19 foi de 49,5%. Gradientes crescentes foram encontrados para a idade e decrescentes por grau de instrução, em comportamento semelhante à mortalidade por esse agravo^8^.

A COVID-longa foi identificada em 32% dos indivíduos que tiveram COVID-19 confirmada, ou seja, em 12,8% do total de participantes, número próximo à estimativa de 10% citada na literatura^27^. Associada às condições de saúde preexistentes, como a autoavaliação de saúde não boa e o diagnóstico anterior de alguma DCNT, a COVID-longa trouxe sequelas importantes por grande período, que provocaram limitações das atividades habituais e afetaram a qualidade de vida, como evidenciado no Brasil e em outros países^28,29^.

A prevalência de COVID-longa estimada no inquérito foi maior entre as mulheres e entre os indivíduos com DCNT, tal como referido em artigo realizado no sul do Brasil^30^. Os achados do presente artigo mostraram que ter tido COVID-longa foi associado à necessidade de internação, em concordância com os achados de outro estudo brasileiro^31^.

As dificuldades financeiras, assim como a insegurança alimentar, foi um elemento importante na morbidade por COVID-19. Durante a pandemia, houve perdas importantes na renda familiar e na situação de trabalho^1^ que, provavelmente, ainda não foram totalmente recuperadas. Adicionalmente, os resultados desta pesquisa mostraram grandes desigualdades socioeconômicas para todos os indicadores de morbidade por COVID-19 considerados no inquérito. Assim, além do aumento na proporção de pessoas com precariedade das condições de vida durante a pandemia, os indivíduos com dificuldades financeiras foram os que menos se vacinaram e os que tiveram maiores prevalências da infecção por SARS-Cov-2 e de COVID-longa, o que, por sua vez, resultou no aumento da proporção de indivíduos que precisaram de mais cuidados de saúde.

Entre os indivíduos que tiveram COVID-19 confirmada, 5,2% precisaram de internação, sobrecarregando os hospitais e as unidades de tratamento intensivo. Ter tido COVID-longa foi a variável mais associada à necessidade de internação. Esses resultados reforçam a importância de identificar e tratar a COVID-longa e otimizar a alocação de recursos no SUS para ações de cuidados específicos dedicados ao atendimento de COVID-19 prolongada nos serviços públicos de saúde^31,32^.

Em relação ao falecimento de algum morador do domicílio, mostrou-se a associação com os domicílios de menor renda *per capita* e com a maior aglomeração domiciliar, refletindo o impacto desigual da pandemia nas famílias socialmente desfavorecidas brasileiras^33^ e com maior número de moradores por cômodo do domicílio, aumentando o risco de transmissão da infecção por SARS-CoV-2.

Em conclusão, os resultados desta pesquisa mostraram que a situação de saúde no Brasil foi bastante afetada pela pandemia de COVID-19 no que se refere tanto à mortalidade como à morbidade, sobretudo na população mais carente. Os achados revelaram grandes desigualdades socioeconômicas, com as menores proporções de vacinação e as maiores prevalências de COVID-19 entre os indivíduos de menor renda, com maiores dificuldades financeiras e que passam por problemas de insegurança alimentar. A idade avançada e as condições preexistentes de saúde (tal como a presença de alguma doença crônica) interferiram na gravidade da doença e no aumento de internações. Entre os indivíduos que relataram COVID-longa, alguns agravos de saúde permanecem até o presente, como a piora na percepção de saúde e as limitações nas atividades habituais. Nesse contexto, a disponibilidade de informações oportunas no período pós-pandêmico apresentadas neste trabalho é de muita relevância para fornecer subsídios às políticas públicas dirigidas aos cuidados dedicados ao diagnóstico e manejo das complicações relacionadas à COVID-19 no sistema público de saúde.

Entre as principais limitações das pesquisas por amostragem em cadeia pela internet, destacam-se: pessoas sem acesso à internet têm probabilidade zero de serem selecionadas; e indivíduos de baixa escolaridade e idosos têm, frequentemente, muitas dificuldades para preencher o questionário *online*, implicando a interrupção do desenvolvimento das redes. Na amostragem pelo método RDS virtual com recrutamento por pares, uma vez que os participantes são voluntários, nem as probabilidades de seleção nem a taxa de não resposta podem ser estimadas.

Outro problema é a obtenção de uma amostra representativa da população brasileira. Nesse estudo, utilizou-se um procedimento de pós-estratificação baseado nas estimativas populacionais da PNAD Contínua-2022, e as distribuições das variáveis utilizadas ficaram, de fato, muito semelhantes às da Pesquisa Nacional por Amostra de Domicílios, contudo a exclusão de uma variável associada a um dos desfechos considerados no procedimento de pós-estratificação pode causar erros nas estimativas médias desses desfechos.

Diferentemente da pesquisa realizada em 2020 por bola de neve virtual^14^, a inclusão dos pares recrutador-recrutado possibilitou a estimação das variâncias dos indicadores de interesse e dos intervalos de confiança e a realização de testes de hipóteses com maior fidedignidade.
